# Are Individuals Perceived as More Attractive within a Group? A Confirmative Study of Group Attractiveness Effect and the Cheerleader Effect in China

**DOI:** 10.3390/healthcare8030344

**Published:** 2020-09-17

**Authors:** Chuanyu Peng, Yanhui Mao, Stefano Pagliaro, Scott Roberts, Stefano Livi

**Affiliations:** 1 School of Public Affairs and Law, Southwest Jiaotong University, Chengdu 610031, China; pengchuanyu@swjtu.edu.cn; 2 Psychological Research and Counseling Center, Southwest Jiaotong University, Chengdu 610031, China; 3 Department of Neuroscience and Imaging, University of Chieti-Pescara, 66100 Chieti, Italy; s.pagliaro@unich.it; 4 Department of Positive Developmental Psychology, Claremont Graduate University, Claremont, CA 91711, USA; scott.roberts@cgu.edu; 5 Department of Social and Developmental Psychology, Sapienza University of Rome, 00185 Rome, Italy; stefano.livi@uniroma1.it

**Keywords:** physical attractiveness, GA-effect, cheerleader effect, selective attention

## Abstract

The stereotype “what is beautiful is good” suggests that having an attractive physical appearance is very important to people’s lives. Physical attractiveness, as an important personal trait, plays vital roles for shaping one’s first impression, and for influencing one’s subsequent evaluation and selection, based on obvious visual features. However, the contextual difference of the physical attractiveness between a group and its group members has been given less attention. For this reason, Van Osch et al. proposed and tested that the perceived physical attractiveness of a group is greater than the average attractiveness of its members (GA-effect), while Walker and Vul found that an individual as a group member is perceived to be more attractive in group context than in isolation (cheerleader effect). Our present work seeks to replicate these two effects on a sample of 1005 Chinese participants to check whether such effects also exist in China, a collectivist culture. Participants were asked to rate the degree of attractiveness presented in each photo stimuli and in each rating condition, and the results show that: (a) the participants’ ratings of physical attractiveness of a group was significantly higher than the average attractiveness of its members (i.e., replicating the GA-effect); (b) the participants’ ratings of physical attractiveness of an individual was evaluated higher in a group than in isolation (i.e., replicating the cheerleader effect); and (c) the larger the group size, the larger the GA-effect. This validating study may aid in understanding human behavior, judgments, and perceptions according to the GA-effect and the cheerleader effect used in a different context in China.

## 1. Introduction

The “what is beautiful is good” stereotype is powerful and widespread [1]. People who are more attractive may receive greater attention and concern [2], receive better judgment [3]), have more friends [4], a more successful career [5], and a longer life expectancy [6]. An attractive individual is imbued with almost all sorts of positive qualities, especially in western European and northern American cultures [7]. The phenomenon of “what is beautiful is good” has been studied for decades [7,8,9,10,11,12,13]. Physical attractiveness refers to people’s evaluations of individuals’ physical appearances based on aesthetic perspective. However, such evaluation of one’s attractiveness may differ, particularly when one is rated alone in comparison to when one is rated in a group context [14]. Exploring the differences between individual attractiveness and group attractiveness may help deepen the understanding of the formation or consolidation of stereotypes, as maintained by Van Osch and colleagues who proposed that there is a group attractiveness effect (GA-effect), and this effect refers to the fact that a group of persons’ collective physical attractiveness is greater than the average attractiveness of its members [10]. By conducting nine experiments, Van Osch et al. found that group impressions of physical attractiveness are more positive than the average ratings of its group members, irrespective of male, female, or mixed gender groups, and that the effect is medium to large and moderated by group size [10]. Walker and Vul found that people seem more attractive in a group context than in isolation, and such a phenomenon has been coined as the cheerleader effect [11]. Subsequent studies have been focused on exploring the mechanism behind the cheerleader effect, and to check the generalization of such an effect [15,16,17,18]. Both attractiveness effects, either the GA-effect or the cheerleader effect, seem to suggest that people in a group context enjoy higher attractiveness rating, irrespective of being rated alone or by comparison to other members within the group.

However, as these findings are mostly based on Western culture, whether such findings are fit for other cultures needs further exploration, since less attention has been devoted to the examination of the GA-effect [12] and the cheer-leader effect [14] in other cultural contexts.

After a quick review of recent studies, we found that in a Korean cultural context, the cheerleader effect was replicated [13,19]. Although the study presented a number of interesting hints about possible replication of the group attractiveness effects, this study is still preliminary as it was presented in a congress and manuscript unpublished, which leaves the question still open. Since Van Osch et al. found group size effect for the GA-effect [10], although Walker and Vul ‘s findings had no such effect for the cheerleader effect [11], we wished to check if individuals are perceived as more attractive in a group context, especially in a collectivist society such as China. Since China has a collectivist culture that emphasizes group cohesiveness, we hypothesize that the evidence for the GA effect and cheerleader effect in collectivist cultures is mixed: as Ojiro et al. failed to replicate the cheerleader effect in Japan, while Eo and colleagues reported a cheerleader effect in Korea [13,19]. The aim of the current study is to investigate whether the GA and cheerleader effects occur in collectivist China, using a well powered experiment. In fact, a sociocultural perspective [20,21] has demonstrated that collectivistic cultures, compared to individualistic cultures, have a different approach to attractiveness, as a result of the role played by group perception on one’s perceived social group identity. Hence, we expect that both cheerleader effect and GA effect are present in China.

Following this research aim, we performed two studies: In Study I, we tested GA-effect in female groups following the design of Van Osch et al. for their “Study 1a”, “Study 1b”, and “Study 2”. We also replicated GA-effect in male groups (“Study 3” by Van Osch et al.) and mixed gender groups (“Study 4” by Van Osch et al.), while tested such effect by within-subject design (“Study 5” by Van Osch et al.) and between-subject design. In Study II, we tested the GA-effect for mixed gender groups (“Study 4” by Van Osch et al.) by within-subject design (“Study 5” by Van Osch et al.). Meanwhile, we also tested the cheerleader effect by comparing the ratings of an individual in group conditions versus isolated conditions. What was different from the replicated studies was that: In Study I, we used Chinese photo stimuli that were rated by Chinese participants, while in Study II we used mixed Chinese and European photo stimuli that were rated by Chinese participants. Possible theoretical explanations as proposed by Van Osch et al. [10], as well as Walker and Vul [11], for supporting such effects are discussed below.

### 1.1. Selective Attention and GA-Effect

Both males and females automatically and intuitively choose to gaze preferentially at attractive faces of other individuals, according to Maner et al. [22]. For this reason, selective attention can play an important role in the study of physical attractiveness: when judging people in terms of a group, people tend to pay more attention to individuals that are most attractive. A common phenomenon in daily life is that attractive individuals may receive longer gaze durations and larger cones of gaze. Scientific evidence, by utilizing eye tracking technique, also finds that more attractive group members receive more visual attention than less attractive group members [10]. Thus, selective attention may lead to the most attractive members of a group exhibiting greater influence on the valuation of the group. For instance, researchers found that people rate faces that they were more familiar with as more attractive compared to those who are not so familiar [23]. Therefore, based on the filter model of selective attention, people’s evaluations of the physical attractiveness of those whom they are most concerned about in a group may influence their valuation of the whole group. If people think highly of someone in the group, they may think highly of their group as a whole, though this effect may be moderated by several factors such as target sex, target baseline level of attractiveness, and the level of attractiveness of the group members [24]. On the contrary, if people are greatly influenced by the least attractive members in the group, they may weaken the evaluation of the whole group [25].

The group attractiveness effect (GA-effect) implies that people perceive groups of individuals as more attractive than the average attractiveness of their members [10]. To support this claim, Van Osch and colleagues conducted nine studies that examined what happens when people evaluate the physical attractiveness of a group compared with the same attractiveness of its group members to test whether the judgments of this attractiveness can be explained by the averaging rule for group impressions [26]. We would expect the evaluation of a group’s physical attractiveness to be based on the average attractiveness of its members [27]. However, in 8 out of 9 studies, Van Osch et al. found that people judge the physical attractiveness of a group to be more attractive than the average of its members. Although the GA-effect was originally found in European culture, its generalization is worth testing—especially in an Eastern culture such as China—since follow-up studies disclosed that a group’s facial attractiveness is indeed the average attractiveness of its members [13].

### 1.2. Cheerleader Effect

The cheerleader effect was popularly mentioned in an American TV show named *How I Met Your Mother* [28], and it refers to a phenomenon that a girl will look more attractive if she is in a group of girls rather than in an isolated situation. To test the existence of the cheerleader effect, Walker and Vul [10] presented the individual’s facial image in a group and in isolation, and found that an individual’s face was rated as more attractive in a group than in isolation, thus confirming the cheerleader effect in a laboratory setting [11]. Ojiro et al. replicated the design of the Experiment 4 conducted by Walker and Vul, and they found the cheerleader effect was not significant in a Japanese culture based on very small size effect [18]. Then, a group of psychologists [15,16] investigated whether the spatial arrangement of the faces in a group would influence the magnitude of the cheerleader effect. Their findings demonstrated that faces were again rated as significantly more attractive in each group configuration than in isolation, and the spatial location of the target face did not influence the size of the cheerleader effect. Thus, the results supported the cheerleader effect in Australia, another individualistic culture, and confirmed that cheerleader effect is a robust phenomenon. A very recent work offered limited evidence for the role of hierarchical encoding in the cheerleader effect and continued to support the cheerleader effect [16]. Ying and colleagues provided evidence that supports the ensemble averaging in facial attractiveness, since one’s brain automatically averages each individual’s facial attractiveness together to create the gist of the collective attractiveness. This confirmed the robustness and replicability of the friend effect in the cheerleader effect, whereby simply being in the company of friends makes an individual look more attractive in a group. Similarly, if the viewer has prior exposure to unattractive groups of faces, then a face will again seem more attractive when subsequently viewed by comparison [14].

### 1.3. Overview of the Present Work

Two studies were conducted to replicate GA-effect and cheerleader effect. Specifically, in Study I, three sub studies were conducted based on 11 sets of photo stimuli, in order to replicate the original studies by Van Osch and colleagues (“Study 1a”, “Study 1b”, and “Study 2” for female groups, “Study 3” for male groups, “Study 4” for mixed gender groups, and “Study 5” for within-subject design). We also tested the cheerleader effect using same sample in Study Ia, Ib, and Ic.

Study Ia was a between-subject design, in which participants enrolled in a university campus were randomly and evenly assigned to three different conditions: in the group condition, they were asked to rate a group as a whole regarding its attractiveness (GR condition); in the other two conditions, they were required to respond to the attractiveness of each single individual (IR condition), or to rate the attractiveness of an individual while they were seen within the group (GMR condition).

Study Ib was a within-subject design, in which university students were called to respond to attractiveness ratings based on GR, GMR, and IR conditions.

Study Ic was again a within-subject design, however, the participants were organizational workers.

Study II aimed to replicate only the GA-effect by a within-subject design based on six groups of photo stimuli with only faces, following the design of the original studies conducted by Van Osch and colleagues (“Study 4” for mixed gender groups, and “Study 5” for within-subject design), in order to eliminate other outlying factors (hip–waist ratio) that may affect the attractiveness rating. Participants were asked to rate a group as a whole in the GR condition, and subsequently rate the attractiveness of each individual while they were seen within the group (GMR condition). Furthermore, a group size effect on the different types of attractiveness ratings was examined.

## 2. Study I

### 2.1. Sample, Materials and Methods

#### 2.1.1. Sample

A total number of 813 subjects were enrolled in Study I. Specifically, in Study Ia, 522 university students (236 males, 286 females) aged between 18 and 27 (*M* = 20.2, *SD* = 2.6) were randomly and evenly assigned to each of the following conditions: the group rating (GR) condition; the group member (GMR) rating condition, and the individual rating (IR) condition; thus, Study Ia was a between-subject design. Study Ib was a within-subject design, with 155 first-year university students (60 males, 95 females) aged between 18 and 20 (*M* = 18.6, *SD* = 0.8) enrolled to judge the attractiveness in sequence for GR, GMR, and IR conditions. Study Ic was also a within-subject design, however, instead of the university students’ sample, 136 organizational workers (76 males, 60 females) were enrolled in the MPA program at Southwest Jiaotong University, China, so that the varying ages (*M_age_* = 36.6, *SD* = 9.8) with different professional backgrounds were considered to cover a broader sample of ages and professions. As Study I did not include any clinical data collection nor pose any psychological harm to participants, the ethics committee of the Southwest Jiaotong University did not demand an ethical application from our project conducted among an adult population. Still, both oral and written informed consent was obtained from the participants prior to our survey and they were assured that participation was anonymous and the data usage was for scientific purposes only.

#### 2.1.2. Study Design and Procedure

**Figure 1:**
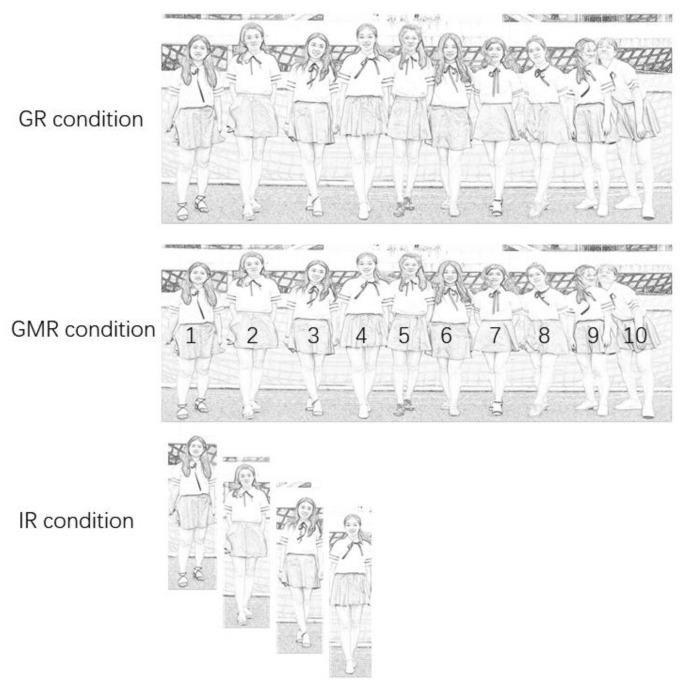
Sample photo stimuli for Study I.

The photo stimuli were provided by 75 university students who were active in the online public social network platform named *Wechat*. In order to ensure the diversity of appearance, features such as varied gender, with or without glasses, hair length, and different facial expressions were considered in the selection criteria, and they were chosen with relatively high, medium, and low attractiveness (pilot tests of one-way ANOVA with attractiveness factor at high, medium, and low levels) with significant difference (all *p*s < 0.05) among each comparison of the attractiveness. The photo stimuli were assigned into three groups which represented three different rating conditions. Specifically, in the GR condition, several individuals appeared together in one photo stimuli in order to be rated as a whole group; in the GMR condition, several individuals’ appearances synthesized together in a single photo stimulus with their corresponding Arabic number for indication (see Figure 1); and in the IR condition, where each individual was presented in a single photo stimulus.

All the selected photo stimuli were obtained with the owner’s informed consent, and they were used in the following three experimental conditions. In the IR condition, the participants were asked to rate each individual photo based on the subject’s attractiveness. In the GMR condition, the participants were asked to locate a specifically targeted person according to the Arabic number among a group of individuals within one photo stimuli (see Figure 1). In the GR condition, the participants were asked to rate the overall attractiveness of a photograph within which several individual members were synthesized together. All photo stimuli were presented in the online platform named Qualtrics Survey Software, by creating survey links based on different rating conditions.

First, all photo stimuli for each rating conditions were separately prepared and presented in each questionnaire, thus three online questionnaires were created. Secondly, three online survey links were prepared by putting these questionnaires on the web. The questionnaires were then administered by sending the links to the potential participants in exchange for researchers’ course credits at *Southwest Jiaotong University*. It was worth noting that the questionnaire was comprised of two parts: the first part contained questions about the participants’ social demographic background, and the second part was a set of questions asking the attractiveness of each presented photo stimuli, based on a 10-point Likert-type scale (i.e., how attractive do you find this person? From 1 = *not at all attractive*, to 10 = *very attractive*). Specifically, the example photo stimuli (for illustration purpose) regarding each condition is indicated in Figure 1.

#### 2.1.3. Measures

In the GR condition, the participants were asked to rate a total of 11 group photo stimuli with a varied number of displayed persons, from 3 to 11 (See Table 1). The participants were asked to respond the attractiveness of the group, considering the following criteria or indicators such as: (1) the physical attractiveness (how pretty the people in the photograph were), (2) friendliness (how kind the people in the photograph seemed), and (3) social attractiveness (to what extent would you want to belong with these people). Then they were required to indicate the degrees of attractiveness of the group (1 = *not at all attractive*, 10 = *very attractive*).

In the GMR condition, the participants were required to evaluate the physical attractiveness of each person shown simultaneously in the single photo set, and each member in the group photo had an Arabic number at the top of their head in order to facilitate distinction. A total of 11 group photo sets that were displayed in GR condition were shown in the experiment, among which 5 were female group photo sets, 4 were male group photo sets, and 2 were mixed-group photo sets. For each photo, participants were asked to rate the following question: how attractive do you find group member X? (1 = *not at all attractive*, 10 = *very attractive*).

In the IR condition, the participants were asked to rate each individual photo that contained only a single person, and a total of 21 personal profile photos were shown. These photos were taken from 3 groups of photo sets among the photo stimuli in two previous conditions. For each photo sets, the participants were asked to answer the questionnaire items based on a 10-point Likert-type scale (i.e., how attractive do you find this person? From 1 = *not at all attractive* to 10 = *very attractive*).

#### 2.1.4. Data Analysis

The questionnaire was distributed and returned through the researchers’ network, because having a convenient sampling improved the flexibility and ability of respondents to accept the survey, and facilitated the diffusion of the questionnaire. Data gathering lasted for 4 months, and data analysis was conducted in SPSS (25.0, IBM, Armonk, NY, USA) for analysis of variance (ANOVA).

### 2.2. Results

#### 2.2.1. Was the Group Attractiveness Equal to the Average Attractiveness of Its Members (GR vs. GMR)? Validation of the GA-Effect

For Study Ia, a between-subject design, we conducted the univariate analysis of variance (ANOVA) with rating condition as independent variable (2 levels), while rating scores as dependent variable. Prior to conducting a series of follow-up ANOVA analyses, the homogeneity of variance assumption was tested for all 11 photo stimuli. Based on a series of Levene test, the homogeneity of variance assumption was considered satisfied (with *p* > 0.05), as reported in Table 1. The group was perceived as more attractive than the average attractiveness of each group member, across each photo stimuli, and with no exception of any single significance level (with one *p* < 0.001, four *p*s < 0.005, six *p*s < 0.05). The partial eta squared (η*_p_*^2^) for measuring the size effect based on the cut-off points for small (0.02–0.12), medium (0.13–0.25), and large effects (>0.26) according to Cohen [29], indicated an overall small effect, with six cases around η*_p_*^2^ = 0.02, which generally confirmed the group attractiveness effect.

Instead of testing the between-subject effect on GA-effect in Study Ia, assigning different university students to rate different rating conditions, we conducted Study Ib adopting a within-subject design by asking another group of university student to rate each of the three conditions randomly. One-way analysis of variance (ANOVA) was adopted to compare the difference between the group ratings and group member ratings. The homogeneity of variance assumption for all 11 photo stimuli was considered satisfactory (with *p* > 0.05) before a series of follow-up comparisons. As indicated in Table 2, the group as a whole was rated to be more attractive than the average of the individual group members, and as such, this difference was significant except in one single case *p* > 0.05 (with five *p*s < 0.001, three *p*s < 0.005, two *p*s < 0.05). The partial eta squared (η*_p_*^2^) for measuring the size effect indicated an overall small effect (with one case of medium effect at 0.127), based on the criteria, the results generally confirmed the GA-effect.

Both Study Ia and Study Ib focused on the university sample, thus in Study Ic, we enrolled a group of 136 organizational workers from different professional backgrounds. We conducted one-way ANOVA with rating condition as a two-level factor (1—GMR condition, 2—IR condition), and rating score as dependent variable, on the premise of the satisfied homogeneity of variance assumption (*p* > 0.05). As indicated in Table 3, the individuals were perceived more attractive when they were in the group context than in the group member context, and this difference was significant with the majority of *p*s < 0.005 (only one case *p* > 0.05). The partial eta squared (η*_p_*^2^) for measuring the size effect based on the criteria of 0.02–0.12, 0.13–0.25, and above 0.26 which are cut-off points for small, medium, and large effects according to Cohen [29], showing an overall small effect, generally supporting the GA-effect.

#### 2.2.2. Were the Individuals Being Perceived as More Attractive When They Were in Group (GMR vs. IR)? Validation of the Cheerleader Effect

To test whether there is any difference on the attractiveness rating between the individual and that individual within a specific group, a series of one-way analysis of variance were carried out to compare the difference of IR and GMR ratings. The homogeneity of variance assumption was satisfied (*p* > 0.05) prior to conducting a series of follow-up ANOVA analyses. The results revealed in Table 4 shows that individuals were rated as more attractive when they were placed in a group, and most differences were observed to be significant (the majority of *p*s < 0.05, with three exceptions *p*s > 0.05). The partial eta squared (η*_p_*^2^) for measuring the size effect indicated a small effect (with fourteen cases) medium effect (with five cases) to large effect (with two cases), based on the cut-off criteria, thus confirming the cheerleader effect.

A series of one-way analyses of variance, with rating conditions as the independent variable and rating scores as the dependent variable, were conducted to compare the difference between group member rating and individual rating, under the satisfactory compliance of the homogeneity of variance assumption (*p* > 0.05). As indicated in Table 5, the group member ratings of attractiveness were greater than the individual ratings, and this difference was significant with the majority of *p*s < 0.001 (with fifteen *p*s < 0.001, two *p*s < 0.005, two *p*s < 0.05, when with the other two cases *p*s > 0.05). The partial eta squared (η*_p_*^2^) for measuring the size effect indicated a small to medium effect (except one case 0.000) based on the cut-off value. Therefore, the individuals were perceived as more attractive than in the group member context, which again confirmed the cheerleader effect.

The previous two pools of university samples supported the cheerleader effect, with the inclusion of another 136 workers which were recruited for rating the individual’s attractiveness within a group or alone. A series of one-way ANOVA were conducted based on the satisfying homogeneity of variance assumption (*p* > 0.05). The results in Table 6 indicate that individuals were perceived as more attractive when they were rated in the group member context rather than alone; and this difference was significant with the majority of *p*s < 0.001 (with one case close to the borderline *p* = 0.08). The partial eta squared (η*_p_*^2^) for measuring the size effect indicated a small to medium effect based on the criteria, and thus mainly supported cheerleader effect.

#### 2.2.3. Was There Any Size Effect on Group Physical Attractiveness?

The results for testing size effect as indicated in Table 7 revealed that there was a significant size effect in the group effect (*p* < 0.001), when the number of individuals increased from three in group photo set P5 to eight in group photo set P6, both GR rating and GMR rating increased and both significantly differed (*p* < 0.005). Specifically, overall, the same individual stimulus was rated higher in a larger size group photo set in comparison to a smaller size group photo set (though with one case such difference level *p* > 0.05). For instance, when the first individual in group photo set P7 was put in a larger size group in P8, she was perceived as more attractive than in a smaller group.

## 3. Study II

### 3.1. Materials and Methods

#### 3.1.1. Sample

A total of 500 Chinese participants were contacted for Study II, of which 399 responded, excluding those who were inattentive or for whom there were missing data on crucial study variables. In total, 192 participants were retained with ages ranging from 20 to 48; of these 37.5% were male, and 80% held a university degree. Such a convenient sample improved the flexibility and capability of respondents to accept the survey, due to the distribution of the questionnaire among researchers’ networks. The participants’ oral and written informed consent was obtained before the experiment, and their participation was anonymous. In addition, the ethics committee of the Southwest Jiaotong University does not demand ethical application from projects conducted among an adult population, which does not include any clinical data collection nor pose any psychological or physical harm to the individuals.

#### 3.1.2. Materials, Study Design and Procedure

**Figure 2:**
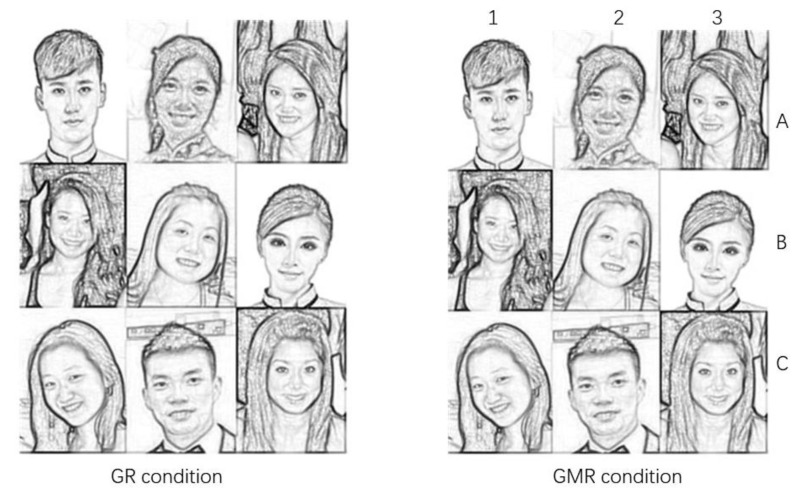
Example photo stimuli for Study II.

Since using the whole body (compared to just the face) in the photo stimuli could cause confounding effects for attractiveness rating, following Study 6 by Van Osch et al. [10], we restricted attractiveness within the faces in Study II to replicate the GA-effect. In order to ensure diversity in appearance, facial features such as glasses, hair length, and skin color were considered in the selection criteria, and the 54 portrait facial photos were all arranged in groups of nine with a combination 3 × 3 matrix. For illustration purposes, example photo stimuli as study materials are depicted in Figure 2. Each photo comprises a mixed gender of both Asian and European adult faces to ensure the integration of various facial features.

We combined each individual face and synthesized 9 of them together in a single group photo. In other words, photo stimuli were presented to the participants in groups of nine which were distributed as a 3 × 3 matrix by a combination of English letter (A–C) and Arabic number (1–3). The participants were asked to respond to questions which were then divided into two parts. Participants were first asked to rate the group’s overall facial attractiveness of the photo stimuli in GR condition (see Figure 2: GR condition), considering the following criteria or indicators such as: (1) physical attractiveness (how pretty the people in the photograph were), (2) friendliness (how kind the people in the photograph seemed), and (3) social attractiveness (to what extent would you want to belong with these people). Then, they were required to indicate the degrees of attractiveness of the group (1 = *not at all attractive*, 10 = *very attractive*): *How attractive do you find this group* (1 = *not at all attractive*, 10 = *very attractive*)? After this, they were asked to rate the attractiveness of each individual face in GMR condition (see Figure 2: GMR condition): *How attractive do you find group member A1* (1 = *not at all attractive*, 7 = *very attractive*)? Thus, Study II was a within-subject design with rating conditions as a factor (two levels with respect to two conditions), where rating scores were the dependent variables.

#### 3.1.3. Data Analysis

Data collected from the online Qualtrics software platform were loaded into the SPSS (25.0) for further analysis. Analysis of variance with rating conditions as a factor (2 levels: GR vs. GMR), and rating scores as dependent variables were applied for comparing the mean differences between the groups.

### 3.2. Results

Was group attractiveness greater than the average attractiveness of its members (GR vs. GMR)? One-way ANOVA was adopted for the difference of group rating and group member rating, based on having homogeneity of variance assumption satisfied (*p* > 0.05). As indicated in Table 8, overall, the group was perceived as more attractive than that attributed by the constitutive group members, and this difference was significant for 5 photo sets with all *p*s < 0.05, with one photo set at the borderline (*p* < 0.069). The partial eta squared (η*_p_*^2^) for measuring the size effect indicated a small to medium effect based on the criteria, thus confirming that the GA-effect was consistent with previous findings by Van Osch et al. [10].

## 4. Discussion

By investigating Chinese participants’ evaluations on physical attractiveness, StudyI expanded the research on GA-effect to another cultural context, and confirmed previous findings on the higher perceived attractiveness of a group compared to the attractiveness of its individual members [10], as well as the higher attractiveness of an individual within a group context rather than in isolation [11,14]. Study II again confirmed the existence of GA-effect by investigating Chinese participants’ evaluations on both Chinese, Korean, and European facial photo stimuli, which was distinct with prior findings obtained in Korea in that the group attractiveness are an average [13]. In general, our main findings are: (a) the group’s physical attractiveness is significantly greater than the average attractiveness of its members, which confirmed the GA-effect; (b) an individual is more attractive in a group setting compared to when he or she is seen alone, which conformed the cheerleader effect; and (c) the larger the group size, the greater the GA-effect.

To illustrate our findings on the GA-effect, the filter model of selective attention [30,31] may help explain why people are motivated to selectively be attracted to the most charming members: as people were faced with a large amount of information, they would selectively receive parts of the information that pertained to their interest. Particularly, in keeping with the “what is beautiful is good” stereotype, people tended to focus on the most attractive members of a given group. Therefore, when people judged the physical attractiveness of a group, they were more likely to be influenced by the most appealing members of that group, making the most attractive individuals account more for overall group attractiveness, thus increasing the group attractiveness [32]. This seemed to confirm that the obsession with beauty or attractiveness is not unique to modern Western culture, but can be also found in Eastern cultures, or even around the world in almost all societies that have been studied [33]. Moreover, when rating attractiveness, we found that in the experimental procedure, no matter which order the participants rate on attractiveness-, GMR first as in Study Ib and Study Ic, or GR first as in Study II-, the GA-effect was consistently found. This reflected on the fact that ratings of the group attractiveness were always higher than the attractiveness of its individual members, thus fully supporting the existence of the GA-effect.

By replicating Walker and Vul’s work, the results generated from Study Ia, Ib, and Ic demonstrated that individuals give higher attractiveness ratings when targets are in group member condition (GMR) in comparison to the individual rating condition (IR). Such a result confirmed the cheerleader effect [11] and was consistent with very recent studies [14,15,16,17,24], therefore extending this finding to a collectivistic culture with a stronger effect size compared to previous studies [18]. According to the explanations proposed by Walker and Vul [11], when rating attractiveness: (1) people’s visual system would automatically compute ensemble representations of faces presented in a group; (2) making the individual members of the group biased toward this ensemble average; and (3) average faces were more attractive. Thus, individuals are rated as more attractive in group based on the interplay of these three cognitive phenomena. Findings that individuals were perceived as more attractive in group member context than in isolation from present dataset supported the cheerleader effect [11], irrespective of an individual that is presented via body image in Study I or via facial image in Study II. As people’s evaluations are influenced by the environmental context and/or other group members, such as ecological conditions may influence one’s judgment of physical attractiveness [27]. When referring to a person’s attractiveness, participants preferentially pay more attention towards the interested target’s attractiveness, automatically integrating the attractive characteristics or stimuli in the group into one average, and this average would affect people’s judgment of group members’ attractiveness. Average characteristics or stimuli were more attractive [23,34], which may have increased people’s rating on group members’ attractiveness leading to individual physical attractiveness garnering higher ratings when in a group, rather than in isolation.

Regarding the effect of the group size on attractiveness, Van Osch et al. [10] found that group size was a significant moderator of the GA-effect, that is, the GA-effect was more likely to be found in larger groups, such as those with six or more members [35]. Our studies, controlling the size of the group, also supported this finding. One possible explanation might be that this is the result of selective attention: as the number of a group increases, people notice more of the objects of interest in that group, which makes more attractive individuals exact much greater influence on the group’s overall judgment, thereby leading to greater deviations from the average attractiveness within larger groups [36]. Another reason might be the result of the priming effect; as Price and colleagues mentioned in previous work, when the number increases, it activates an internal representation of relative quantity that directly affects the internal representations of magnitude, thus in turn affecting judgements of the average [37]. Moreover, consistent with previous studies [35,36,37], our results which were revealed from present datasets could not allow us to draw further conclusions on numbers that could be enough for generating such a size effect.

### 4.1. Implications

Theoretically speaking, the generalization of two previously tested attractiveness effects where either GA-effect or cheerleader effect, are confirmed in a collectivist culture as represented in China. Specifically, both Study I and Study II confirmed previous work on GA-effect within European culture [35] which was different from previous findings obtained in Korea [13]. Study I also confirmed the cheerleader effect that was found in the U.S. [11], consisted with previous work validated in Japan [18], as well as in Australia [15,16]. In this sense, the present work may provide additional evidence for understanding how and why people’s perceptions, judgement, and behaviors can be influenced by attractiveness, irrespective of individual or collectivist culture forms [38]. In a deeper sense, our work added evidence for understanding the selective attention theory, as well as the mechanism of hierarchical encoding in the cheerleader effect.

Practically speaking, the findings yielded from the present datasets suggest that people are more attractive when they are evaluated in the group context, and this result illustrates the importance of the environmental or contextual factors in evaluating physical attractiveness. On one hand, it suggests that choosing a good group may help facilitate self-image and increase attractiveness [39]; on the other hand, it warns us that individual physical attractiveness evaluated in group may be influenced by some other group members within that group, so we should be more careful when making judgments about people’s physical attractiveness, whether alone or in groups. Secondly, the overall group physical attractiveness is greater than the average attractiveness of the group members, suggesting that people may selectively pay attention to someone in the group that they find more attractive, and such attention and preference to attractive individuals will affect people’s evaluations of the whole group. Evidence found that when rating the attractiveness of romantic partners, being in a group context increased the attractiveness of an individual when they met the minimum level of attractiveness of the group, which comprised of some highly attractive individuals [24]. For example, in commercial advertisement, the adoption of a group of individuals as advertising spokespersons may be more likely to be noticed and welcomed by consumers; in leisure activities such as singing shows or talent shows, the inclusion of celebrities may be more likely be appreciated and viewed by the audience. Men across cultures place greater importance than women do on physical attractiveness of a prospective partner, choosing a group in which one’s attractiveness can be increased seems to be a good mating strategy for women [14,40]. As the size of the group increase, the larger the GA-effect, suggesting that the number of team members may affect the physical attractiveness of the overall team, as well as each member in this team. For example, in the business and management negotiation, the selection and combination of a team with more attractive members may increase the partner’s evaluation of the whole team of that company and help achieve the negotiation agreement. In general, the attractiveness effect, where either GA-effect or cheerleader effect, seems widespread in our daily lives, not only affecting our judgment of people’s physical attractiveness, but more importantly, perhaps being an important basis for our judgment and decision-making in many walks of life. Previous works speculated that the GA-effect and the cheerleader effect might influence the above-mentioned situations, but there is no evidence yet. Therefore, this confirmative study could help us understand human behavior, judgments, and perceptions over the attractiveness effects used in different contexts and across cultures [41].

### 4.2. Limitations and Future Research

This paper replicated some of the studies by Van Osch and colleagues in order to investigate whether the group attractiveness effect is detectable in a non-European country such China. Our results confirmed the existence of such a GA-effect in Chinese context through two sub-studies. The present work also validated the cheerleader effect in Chinese context. However, a few limitations warrant notice. First, following the design of the original study, we neglected the fact that some of the ratings of physical attractiveness of an individual may not only be based on facial attractiveness, but also on body height or body shape or clothing, or even age or ethnicity which are known to influence participants’ perceptions, and in this sense we applied only faces in Study II to rule out above mentioned influential factors. Second, although we recruited both university students and real workers, this sample size may be not large enough to represent the whole Chinese population, thus it may affect the generalization of our findings. Also, regarding an eventual gender effect, the present sample failed to test the influence of the participant’s gender difference in various ways of rating time length of participants’ attention to the stimuli, their eye-tracking, or other factors on GA-effect as in study by Van Osch et al. Thus, future work may continue analyzing the gender effect to compare male and female attractiveness within larger sample sizes, as well as that of attractiveness being rated by the same or opposite genders. Future work may also continue to study different sample groups to determine the attractiveness ratings based on different ethnicities, Chinese stimuli versus foreign (i.e., European) stimuli. Finally, in terms of tools and instruments, both qualitative and quantitative accounts including additional physiological data might be gathered to ensure understanding of these effects. Possible causes and effects of GA-effect intuitively and accurately facilitate application of the GA-effect in the fields of everyday life, such as advertising, entertainment, management negotiation, and so on, as possible research directions for future work.

## 5. Conclusions

Two studies replicated the GA-effect and the cheerleader effect. Overall, our findings confirm that the GA-effect is detectable in a collectivistic cultural context in China, that is to say, the perceived physical attractiveness of a group was greater than the average attractiveness of its group members [10], and the perceived physical attractiveness of an individual was greater in the group context than in isolation [11]. It seems that group context may help yield advantages for increase of people’s attractiveness, however, we are not sure if it is the factor of collectivistic culture that can account for this, due to present datasets. As such, further research may benefit from designing experiments for exploring the underlying mechanism by investigating psychological and neural factors.

## Figures and Tables

**Table 1:** Comparisons between the GR and GMR from Study Ia (*n* = 522).

Photo Stimuli	No. of Person	GR (*n* = 174)		GMR (*n* = 174)		95% CI	*F*	*p*	η*_p_*^2^
		*M*	*SD*	*M*	*SD*				
**P1**	10 (all females)	5.47	2.04	4.70	2.28	[0.24, 1.30]	10.94	0.001	0.031
**P2**	7 (all females)	5.60	1.89	5.10	2.04	[0.06, 0.94]	5.67	0.018	0.016
**P3**	5 (all females)	6.56	2.09	5.98	2.07	[0.06, 1.11]	6.86	0.009	0.019
**P4**	6 (all females)	5.26	1.49	4.64	2.62	[0.09, 1.15]	7.38	0.007	0.021
**P5**	3 (all males)	4.59	1.69	4.07	2.53	[0.07, 0.96]	5.03	0.026	0.014
**P6**	8 (all males)	5.05	1.84	4.31	2.50	[0.29, 1.19]	9.92	0.002	0.028
**P7**	4 (all males)	4.73	2.50	4.14	2.36	[0.07, 1.11]	5.16	0.024	0.015
**P8**	11 (all females)	5.02	2.11	4.29	2.43	[0.24, 1.21]	8.82	0.003	0.025
**P9**	6 (4 females, 2 males)	5.44	2.18	4.87	2.24	[0.11, 1.04]	5.85	0.016	0.017
**P10**	6 (4 females, 2 males)	5.83	2.18	5.14	2.14	[0.22, 1.17]	8.97	0.003	0.025
**P11**	4 (2 females, 2 males)	5.69	2.17	4.69	2.24	[0.52, 1.49]	17.98	0.000	0.049

**Table 2:** Comparisons between the GR and GMR from Study Ib (*n* = 155).

Photo Stimuli	No. of Person	GR		GMR		95% CI	*F*	*p*	η*_p_*^2^
		*M*	*SD*	*M*	*SD*				
**P1**	10 (all females)	5.60	1.88	5.05	2.04	[0.02, 1.09]	33.74	0.041	0.127
**P2**	7 (all females)	5.66	2.12	4.79	1.95	[0.41, 1.32]	14.06	0.000	0.044
**P3**	5 (all females)	6.74	1.98	6.08	1.92	[0.14, 1.19]	7.67	0.001	0.032
**P4**	6 (all females)	5.21	1.51	4.93	2.04	[0.23, 0.78]	28.58	0.000	0.110
**P5**	3 (all males)	4.50	1.72	4.01	2.22	[0.05, 0.94]	4.77	0.030	0.015
**P6**	8 (all males)	4.53	1.71	3.92	2.10	[0.18, 1.04]	7.86	0.005	0.025
**P7**	4 (all males)	4.52	1.80	3.47	1.95	[0.63, 1.47]	24.25	0.000	0.073
**P8**	11 (all females)	4.54	1.83	3.83	2.14	[0.26, 1.15]	9.77	0.002	0.031
**P9**	6 (4 females, 2 males)	5.31	1.90	4.76	2.14	[0.09, 0.96]	5.62	0.018	0.037
**P10**	6 (4 females, 2 males)	5.65	1.92	4.83	1.85	[5.35, 5.96]	14.88	0.000	0.094
**P11**	4 (2 females, 2 males)	6.03	1.88	5.54	1.86	[0.10, 0.90]	5.44	0.200	0.038

**Table 3:** Comparisons between the GR and GMR from Study Ic (*n* = 136).

Photo Stimuli	No. of Person	GR		GMR		95% CI	*F*	*p*	η*_p_*^2^
		*M*	*SD*	*M*	*SD*				
**P1**	10 (all females)	6.48	1.85	5.71	2.19	[0.29, 1.25]	40.20	0.002	0.035
**P2**	7 (all females)	6.64	1.75	5.96	2.02	[0.23, 1.13]	8.89	0.003	0.032
**P3**	5 (all females)	7.15	1.77	6.63	2.06	[0.07, 0.98]	17.88	0.025	0.018
**P4**	6 (all females)	5.90	2.04	5.79	2.31	[−0.21, 0.83]	27.04	0.240	0.005
**P5**	3 (all males)	5.69	1.92	4.76	2.74	[0.37, 1.50]	10.61	0.001	0.038
**P6**	8 (all males)	5.96	1.91	4.82	2.66	[0.59, 1.70]	16.69	0.000	0.058
**P7**	4 (all males)	5.96	1.99	4.69	2.49	[0.64, 1.81]	21.73	0.000	0.074
**P8**	11 (all females)	6.05	1.90	4.85	2.71	[0.64, 1.76]	17.76	0.000	0.062
**P9**	6 (4 females, 2 males)	6.09	1.84	5.57	2.16	[0.74, 0.99]	4.46	0.036	0.016
**P10**	6 (4 females, 2 males)	6.76	1.68	5.86	1.97	[0.04, 1.33]	16.37	0.000	0.057
**P11**	4 (2 females, 2 males)	6.48	1.98	5.53	2.16	[0.45, 1.44]	14.16	0.000	0.050

**Table 4:** Comparisons between GMR and IR from Study Ia.

Photo	No.	GMR (*n* = 174)		IR (*n* = 174)		95% CI	*F*	*p*	η*_p_*^2^
		*M*	*SD*	*M*	*SD*				
**P1**	1	4.26	2.71	3.52	2.47	[0.19, 1.28]	7.22	0.008	0.040
	2	5.44	2.50	4.93	2.55	[−0.03, 1.05]	3.52	0.062	0.020
	3	4.82	2.44	4.43	2.53	[−0.14, 0.92]	2.50	0.144	0.012
	4	5.21	2.71	4.12	2.34	[−1.62, −0.55]	16.02	0.000	0.085
	5	4.88	2.59	3.48	2.13	[0.89, 1.89]	30.69	0.000	0.150
	6	4.65	2.56	3.56	2.19	[−1.59, −0.61]	19.72	0.000	0.102
	7	4.36	2.45	3.00	1.89	[0.91, 1.82]	34.81	0.000	0.168
	8	4.14	2.36	3.10	1.95	[−1.48, −0.59]	34.22	0.000	0.165
	9	4.87	2.24	2.62	1.86	[1.82, 2.68]	62.99	0.000	0.267
	10	5.14	2.14	2.29	1.72	[−3.25, −2.44]	85.47	0.000	0.331
**P3**	1	5.84	2.47	5.80	2.40	[−0.48, 0.55]	0.02	0.892	0.000
	2	5.08	2.22	3.96	2.22	[0.65, 1.58]	22.30	0.000	0.114
	3	6.03	2.3	5.88	2.49	[−0.66, 0.36]	0.36	0.552	0.002
	4	6.02	2.48	6.61	2.22	[−1.06, −0.12]	6.10	0.014	0.034
	5	6.91	2.25	6.07	2.23	[0.39, 1.30]	13.46	0.000	0.072
**P4**	1	4.86	2.76	3.64	2.24	[0.68, 1.76]	20.14	0.000	0.104
	2	4.78	2.76	3.67	2.06	[0.60, 1.62]	18.66	0.000	0.097
	3	4.53	2.76	3.31	2.14	[0.71, 1.73]	22.36	0.000	0.114
	4	5.05	2.87	4.05	2.40	[0.44, 1.54]	12.59	0.001	0.068
	5	4.99	2.84	3.64	2.13	[0.83, 1.89]	25.89	0.000	0.130
	6	3.66	2.56	2.47	1.79	[0.76, 1.62]	29.31	0.000	0.145

**Table 5:** Comparisons between IR and GMR from Study Ib (*n* = 155).

Photo	No.	GMR		IR		95% CI	*F*	*p*	η*_p_*^2^
		*M*	*SD*	*M*	*SD*				
**P1**	1	4.52	2.47	3.7	2.19	[0.27, 1.36]	8.68	0.004	0.053
	2	5.74	2.40	5.06	2.42	[0.14, 1.23]	6.11	0.015	0.038
	3	5.48	2.27	4.4	2.17	[0.58, 1.60]	17.58	0.000	0.102
	4	5.24	2.25	4.21	2.52	[0.50, 1.57]	14.49	0.000	0.086
	5	5.22	2.30	3.71	2.12	[0.98, 2.05]	31.16	0.000	0.168
	6	5.27	2.31	3.92	2.13	[0.84, 1.88]	26.63	0.000	0.147
	7	4.71	2.27	3.42	2.12	[0.77, 1.81]	23.90	0.000	0.134
	8	4.77	2.27	3.59	2.06	[0.66, 1.70]	20.34	0.000	0.117
	9	4.81	2.54	3.09	2.13	[1.18, 2.26]	40.06	0.000	0.206
	10	4.73	2.63	3.00	2.26	[1.17, 2.30]	36.35	0.000	0.191
**P3**	1	5.73	2.38	5.67	2.40	[−0.49, 0.60]	0.043	0.837	0.000
	2	5.1	2.28	4.56	2.16	[0.04, 1.05]	4.55	0.034	0.029
	3	6.27	2.33	6.34	2.42	[−0.59, 0.47]	0.058	0.811	0.000
	4	5.83	2.26	7.30	2.42	[−1.96, −0.96]	33.43	0.000	0.178
	5	7.39	2.08	5.84	2.33	[1.05, 2.05]	37.35	0.000	0.195
**P4**	1	5.08	2.33	3.69	2.07	[0.88, 1.90]	29.04	0.000	0.159
	2	4.97	2.35	3.70	2.11	[0.75, 1.78]	23.67	0.000	0.133
	3	4.69	2.38	3.64	2.08	[0.54, 1.56]	16.55	0.000	0.097
	4	5.85	2.26	4.59	2.29	[0.75, 1.79]	23.36	0.000	0.132
	5	5.48	2.31	3.97	2.26	[0.98, 2.05]	31.22	0.000	0.169
	6	3.46	2.27	2.69	1.96	[0.30, 1.23]	10.46	0.001	0.064

**Table 6:** Comparisons between IR and GMR from Study Ic (*n* = 136).

Photo	No.	GMR		IR		95% CI	*F*	*p*	η*_p_*^2^
		*M*	*SD*	*M*	*SD*				
**P1**	1	5.38	2.72	4.32	2.42	[0.44, 1.67]	11.51	0.001	0.041
	2	6.37	2.35	5.30	2.39	[0.51, 1.64]	13.95	0.000	0.049
	3	5.89	2.43	4.94	2.31	[0.38, 1.51]	10.90	0.001	0.039
	4	5.92	2.36	4.68	2.29	[0.68, 1.79]	19.26	0.000	0.067
	5	5.96	2.51	4.03	2.39	[1.35, 2.52]	42.26	0.000	0.135
	6	5.64	2.42	4.38	2.38	[0.70, 1.85]	19.13	0.000	0.066
	7	5.43	2.32	4.01	2.38	[0.87, 1.99]	25.03	0.000	0.085
	8	5.55	2.51	4.22	2.29	[0.76, 1.90]	20.91	0.000	0.072
	9	5.63	2.44	3.64	2.33	[0.76, 1.90]	46.57	0.000	0.256
	10	5.29	2.57	3.40	2.40	[1.28, 2.50]	37.18	0.000	0.216
**P3**	1	6.37	2.33	5.57	2.56	[0.21, 1.38]	7.22	0.008	0.051
	2	5.88	2.36	4.22	2.34	[1.13, 2.20]	37.60	0.000	0.218
	3	6.54	2.36	5.85	2.45	[0.13, 1.24]	6.00	0.016	0.042
	4	6.96	2.22	6.89	2.47	[−0.48, 0.60]	0.06	0.081	0.000
	5	7.41	2.15	6.20	2.30	[0.68, 1.75]	20.01	0.000	0.129
**P4**	1	5.54	2.62	4.06	2.38	[0.89, 2.06]	24.88	0.000	0.156
	2	5.49	2.47	4.18	2.23	[0.79, 1.84]	25.17	0.000	0.157
	3	5.63	2.55	4.10	2.20	[0.97, 2.10]	28.94	0.000	0.177
	4	6.06	2.53	4.86	2.35	[0.63, 1.77]	17.36	0.000	0.114
	5	6.24	2.54	4.47	2.25	[1.20, 2.33]	37.90	0.000	0.219
	6	4.56	2.84	3.11	2.40	[0.83, 2.06]	21.70	0.000	0.138

**Table 7:** Test of size effect on GR, GMR, and IR from Study Ia (*n* = 522).

Photo Set		Bigger Group (*M* ± *SD*)	Small Group (*M* ± *SD*)	95% CI	*t*	*p*
GR	P5_GR_ (*n* = 3) vs. P6_GR_ (*n* = 8)	4.31 ± 2.14	3.85 ± 1.98	[−0.59, −0.33]	7.07	0.000
	P7_GR_ (*n* = 4) vs. P8_GR_ (*n* = 11)	4.57 ± 2.49	4.35 ± 2.35	[−0.41, −0.03]	2.31	0.020
GMR	P5_GMR_ (*n* = 3) vs. P6_GMR_ (*n* = 8)	4.23 ± 2.54	4.04 ± 2.55	[−0.32, −0.06]	2.97	0.000
	P7_GMR_ (*n* = 4) vs. P8_GMR_ (*n* = 11)	4.28 ± 2.54	4.10 ± 2.44	[0.08, 0.27]	3.49	0.000
IR	The 1st person in P5_GMR_ vs. P6_GMR_	4.57 ± 2.81	4.25 ± 2.77	[−0.48, −0.16]	3.98	0.000
	The 2nd person in P5_GMR_ vs. P6_GMR_	4.35 ± 2.73	4.07 ± 2.71	[−0.42, −0.14]	3.92	0.000
	The 3rd person in P5_GMR_ vs. P6_GMR_	4.17 ± 2.63	3.80 ± 2.51	[−0.55, −0.20]	4.2	0.000
	The 1st person in P7_GMR_ vs. P8_GMR_	4.32 ± 2.74	4.18 ± 2.65	[−0.34, 0.06]	1.36	0.180
	The 2nd person in P7_GMR_ vs. P8_GMR_	3.91 ± 2.65	3.66 ± 2.62	[0.09, 0.41]	3.03	0.000
	The 3rd person in P7_GMR_ vs. P8_GMR_	4.71 ± 2.72	4.85 ± 2.91	[−0.08, 0.36]	−1.25	0.210
	The 4th person in P7_GMR_ vs. P8_GMR_	4.36 ± 2.76	4.18 ± 2.72	[−0.34, −0.03]	2.28	0.020

**Table 8:** Comparisons between GR and GMR from Study II (*n* = 192).

Photo Stimuli	No.	GR		GMR		95% CI	*F*	*p*	η*_p_*^2^
**1**	9	4.70	1.46	4.14	0.92	[4.30, 4.55]	20.61	0.000	0.194
**2**	9	4.00	1.55	3.66	1.00	[3.70, 3.96]	6.51	0.011	0.069
**3**	9	4.03	1.47	3.72	0.93	[3.75, 4.00]	6.11	0.014	0.069
**4**	9	4.02	1.31	3.82	0.79	[3.81, 4.03]	3.32	0.069	0.025
**5**	9	4.33	1.29	3.75	1.01	[3.92, 4.16]	24.20	0.000	0.241
**6**	9	4.47	1.47	3.99	0.94	[4.10, 4.35]	14.68	0.000	0.162
